# Validation of spinal motion with the spine reposition sense device

**DOI:** 10.1186/1743-0003-6-12

**Published:** 2009-04-22

**Authors:** Cheryl M Petersen, Peter J Rundquist

**Affiliations:** 1 Concordia University Wisconsin, 12800 North Lake Shore Drive, Mequon, WI 53097, USA; 2 University of Indianapolis, Krannert School of Physical Therapy, 1400 East Hanna Avenue, Indianapolis, IN 46227, USA

## Abstract

**Background:**

A sagittal plane spine reposition sense device (SRSD) has been developed. Two questions were addressed with this study concerning the new SRSD: 1) whether spine movement was occurring with the methodology, and 2) where movement was taking place.

**Methods:**

Sixty-five subjects performed seven trials of repositioning to a two-thirds full flexion position in sitting with X and Y displacement measurements taken at the T4 and L3 levels. The thoracolumbar angle between the T4 and the L3 level was computed and compared between the positions tested. A two (vertebral level of thoracic and lumbar) by seven (trials) mixed model repeated measures ANOVA indicated whether significant differences were present between the thoracic (T4) and lumbar (L3) angular measurements.

**Results:**

Calculated thoracolumbar angles between T4 and L3 were significantly different for all positions tested indicating spinal movement was occurring with testing. No interactions were found between the seven trials and the two vertebral levels. No significant findings were found between the seven trials but significant differences were found between the two vertebral levels.

**Conclusion:**

This study indicated spine motion was taking place with the SRSD methodology and movement was found specific to the lumbar spine. These findings support utilizing the SRSD to evaluate changes in spine reposition sense during future intervention studies dealing with low back pain.

## Background

Patients with low back pain present with impaired spine reposition sense and altered motor control. [1-5] Motor control problems found include a delay in feed-forward control of the transversus abdominis with upper and lower extremity movements within subjects with low back pain compared to controls. [6-8] Also, the loss of multifidus cross sectional area, occurring with the first episode of low back pain, has been improved with biofeedback training with decreased low back pain recurrence rates one, two and three years later. [9-11] However, evaluation of proprioception, as an outcome measure, has not been performed as part of these studies, in spite of suggesting rehabilitation was addressing proprioception.

The clinicians/researchers involved with the development of this new spine reposition sense device (SRSD) have found many devices (piezoelectric accelerometer, [1] Lumbar Motion Monitor, [2] 3SPACE, [12,13] Fastrak [4,14,15] and an ultrasound movement analysis system [16]) used in the literature to measure spine reposition sense. These various devices have not been used in the clinical setting to evaluate spine proprioception nor have they been used as an outcome measure during spine proprioception rehabilitation. It was hypothesized that the cost, lack of ease of use, no metal in the area (3SPACE and Fastrak) or time required to use these various devices, was the explanation for the fact that these devices were not used to demonstrate proprioception change with rehabilitation in low back pain research. Therefore, a device which could be easily incorporated into clinical research or the clinical setting was proposed as necessary. Three phases of research have been carried out with SRSD. The number of trials to test spine reposition sense have been determined, test-retest reliability and validation of the device compared to the Skill Technologies 6D (ST6D) Imperial Motion Capture and Analysis System, have been established. [17] The SRSD methodology [12] involved sitting and reproducing a two-thirds position of full flexion seven times compared to a reference two-thirds position. The X and Y displacement measurements, using trigonometry (theta = tan^-1 ^X/Y), produced angles which can be compared. The device's measurement methodology has been challenged though regarding whether movement in the spine was occurring with reposition sense testing. The flexion motion has been thought to be due to rotation about the pelvis on the femurs and not due to lumbar flexion. Also, measurements have been taken from the T4 level which does not implicate lumbar spine motion with testing.

Trunk range of motion is important to function. Values for trunk flexion range from 51° to 62° (OSI CA-6000 Spine Motion Analyzer data). [18] Trunk movement is essential for the movement of sit-to stand. The propulsive impulse at the beginning of movement initiating forward momentum is thought to be generated by the angular velocity of the trunk and pelvis in the sagittal plane. [19] Average values of 16 degrees of trunk flexion on the pelvis have been found. [20] Differences in subjects with low back pain compared to controls have been found for the rotational relationship between the thorax and the pelvis during gait [21] and intra-subject variability has been noted in pelvic and thoracic angular displacements in subjects with low back pain. [22] A higher stride-to-stride variability in angular displacements was found and may be due to deficits in motor control and spine proprioception.

The purpose, therefore of this study, was to determine if spine movement was present during testing with the SRSD and where in the spine motion was taking place. Movement was suspected in both the thoracic and lumbar areas and the relative amounts in the two regions would be described from measurements taken from the two locations at the T4 and L3 levels.

Movement in the lumbar area should be present to allow further use of the device to examine lumbar intervention(s) proposed to improve spine reposition sense, as suggested in the literature but not measured. Two hypotheses were tested: 1) no difference would be found in the thoracolumbar angle between the various positions tested and 2) no difference would be found between the angular measurements taken at the T4 and L3 locations across the seven trials used in testing.

## Methods

### Subjects

Subjects were recruited on a volunteer basis from a university campus as a convenience sample of 65 adults. Inclusion criteria included ≤ 5% score on the Oswestry Low Back Pain Questionnaire, a lower age limit of 18 years, set to target subjects with a developed proprioceptive system [23,24] and an upper age limit of 40 years, in an attempt to reduce the effect of age-related changes in position sense. [25-30] Exclusion criteria are presented in Table 1, and descriptive statistics for these subjects are presented in Table 2. Informed consent was obtained by all subjects in compliance with both the University of Indianapolis and Concordia University's Human Subject's Institutional Review Board guidelines.

**Table 1:** Exclusion criteria (by self-report)

Oswestry back pain scores of greater than or equal to 5%
Balance, coordination, or stabilization therapy within the last six months

Excessive use of pain medication, drugs, or alcohol

Ligamentous injury to the hips, pelvis, or spine

Spinal surgery

Balance disorders secondary to: active or recent ear infections, vestibular disorders, trauma to the vestibular canals, or orthostatic hypotension

Neurologic disorders including: multiple sclerosis (MS), cerebral vascular accident (CVA), spinal cord injury, neuropathies, and myopathies

Diseases of the spine including: osteoporosis, instability, fractures, rheumatoid arthritis (RA), degenerative disc disease (DDD), and spondylolisthesis

**Table 2:** Descriptive statistics for subject characteristics

**Number**	65
**Age**	
(Mean ± SD)	23.4 ± 2.9

**Sex Ratio**	
Male:Female	14: 51 (27.5%)

**Height **(cm)	
(Mean ± SD) Female, Male	169.1 ± 7.2, 180.5 ± 7.1

**Weight **(kg)	
(Mean ± SD) Female, Male	65.5 ± 10.5, 86.5 ± 14.4

### Protocol

**Figure 1:**
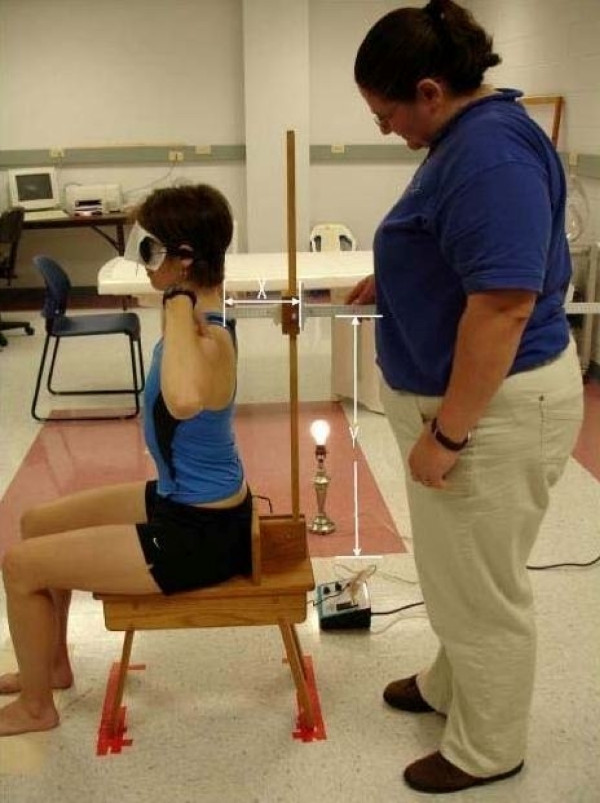
**The measurement method: X and Y coordinates are measured and used in a trigonometric calculation to determine the angle**. An individual is shown seated in the upright neutral posture; during the study, all subjects were blindfolded throughout testing.

**Figure 2:**
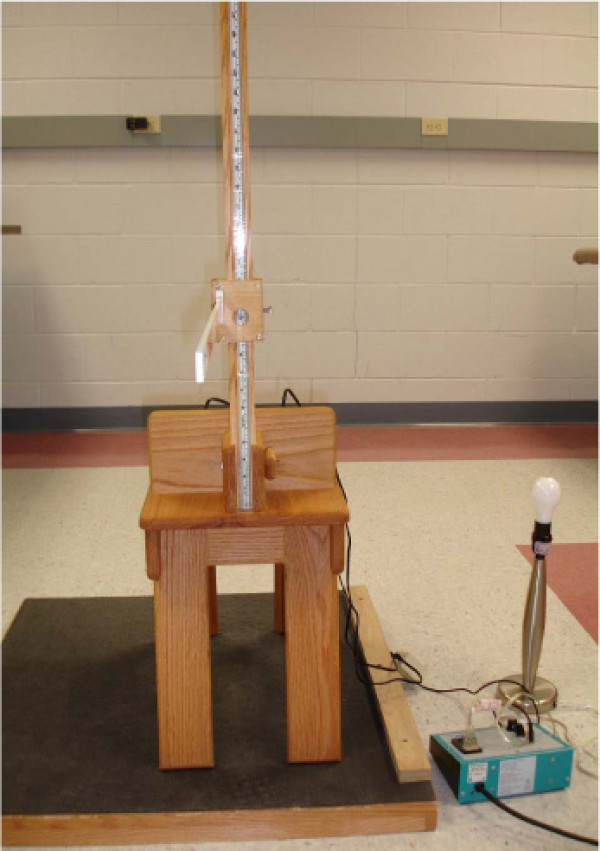
**Vertical measurement view for the new SRSD method taken through an opening in the back of the sliding mechanism**.

**Figure 3:**
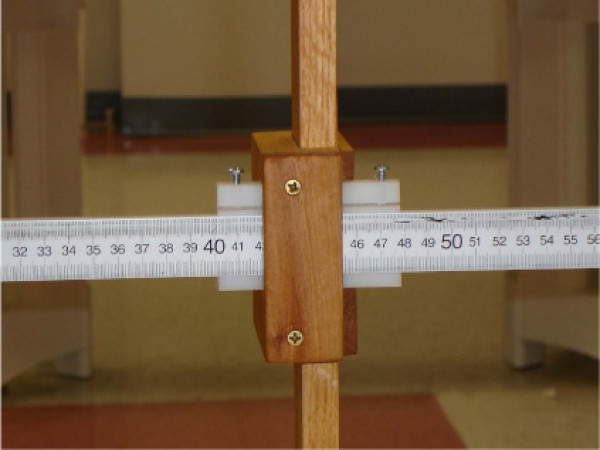
**Horizontal measurement view for the new SRSD method taken from the side of the sliding mechanism**.

The new device consists of two meter sticks and a sliding mechanism. One meter stick is positioned vertically and the second meter stick extends horizontally, perpendicular to the vertical meter stick (Figure 1). The horizontal meter stick has a level attached and the vertical meter stick is perpendicular to a leveled wooden stool, upon which the subject sits. A flat piece of wood (wooden seat back) is bolted to the stool for subjects to place their sacrum and ilia against for positioning. Vertical measurement in centimeters is taken through an opening within the sliding mechanism (Figure 2) and the horizontal measurement is taken from the front of the sliding mechanism in centimeters (Figure 3), measuring the distance from the vertical meter stick to a point over the spine. Leveling the entire device ensures 90° angles, enabling the use of a trigonometric equation in measuring trunk orientation and reposition error. To calculate the angle, the X and Y displacement information is used within the trigonometric equation, theta = tan^-1 ^X/Y (Figure 1). According to previous literature, the range of mean absolute repositioning error (ARE) for flexion movements of the trunk was from 1.67 – 7.1° [2,31-33] and the mean ARE range for the SRSD trials was from 1.84 – 2.68°. The measurement resolution of the new device was determined to be 0.17° (± 1 mm in X and Y). Test-restest reliability of the device over a week's time frame was found to produce similar values using the Bland Altman method which has been suggested in the literature as necessary for repeated trials. [34-36] Validation of the device against the gold standard Skill Technologies 6D Imperial Motion Capture and Analysis System revealed similar measures for the two devices within the sagittal plane using the Bland Altman method [36] and an ICC (3, 1) of 0.99 (CI 0.55, 0.99; SEM 0.47). [17]

**Figure 4:**
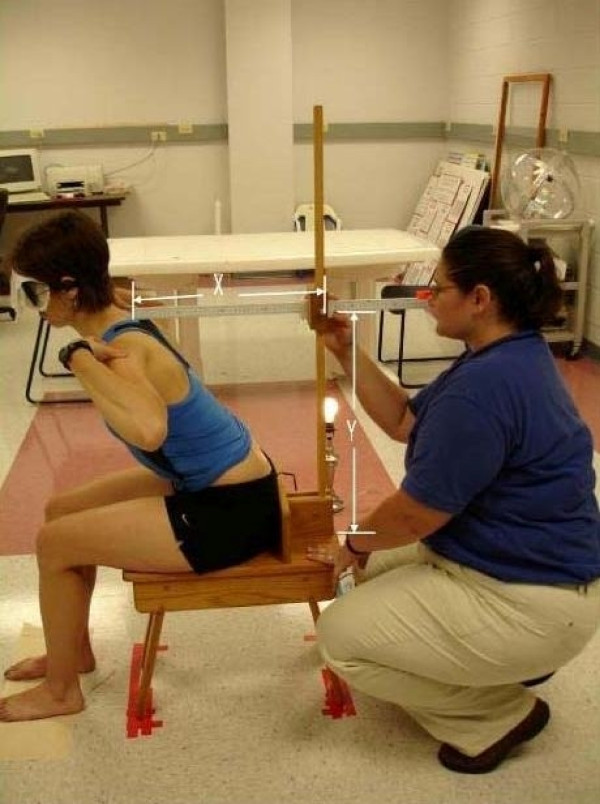
**The measurement method: The X and Y coordinates are shown above with an individual in a position 2/3 of full flexion; during the study, all subjects were blindfolded throughout testing**.

Subjects were tested with measurements taken from both the T4 and L3 levels for all movements prior to any movement change. The protocol (evaluated for the number of repeated trials of flexion repositioning, test-retest reliability and validity of measurements) [17] involved each subject assuming first a neutral position, they then move into as much flexion as they can keeping their sacrum and ilia against the wood piece, next they assume a position that is two-thirds of their full flexion position (Figure 4) and are asked to remember that position. They repeat the two-thirds position for seven trials and last return to their neutral posture. They return to the upright starting posture (Figure 1) following each movement and all movements are tested at one time. To indicate the pelvis was positioned against the wooden seat back suggesting lumbar spine movement was occurring, two sensors (Pal Pad, Adaptivation Incorporated, 2225 West 50^th ^Street, Sioux Fall, SD 57105), attached to PowerLink (LAB Resources, 161 West Wisconsin Avenue, Suite 2G, Pewaukee, WI 53072), activated by 1.2 ounce of pressure, were placed on the wooden seat back 2.5 cm apart to activate a light. If the circuit was broken, the light turned off, and movement away from the wooden seat was indicated. This was considered a mistrial and the pelvis was repositioned for sensor contact and light activation (Figures 1 and 4). Angular measurements were computed using the trigonometric method to determine angular values from the X and Y displacements taken at the two levels.

### Analysis

#### Spine versus hip movement

**Figure 5:**
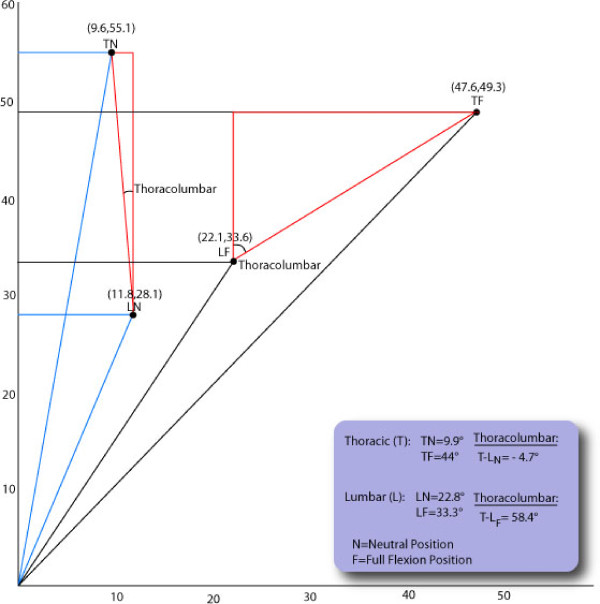
**Illustration of the derivation of the thoracolumbar angle measures where T = thoracic, L = lumbar, N = neutral position, F = full flexion position**.

For the first goal, if the spine was relatively rigid with movement occurring primarily at the pelvis on the femurs, angular measurements would be similar at all positions of testing. We computed the angle above L3 for each movement by using the horizontal and vertical measurements from the thoracic and the lumbar trials. Horizontal X and vertical Y differences were computed respectively by using the thoracic X – lumbar X measurements and the thoracic Y – lumbar Y measurements. These difference measurements for X and Y were then used in the trigonometric equation, theta = tan^-1 ^X_difference_/Y_difference _to calculate the angle occurring between the T4 and the L3 level (the thoracolumbar angle). See Figure 5 for a representative subject's data for two positions, neutral (N) and full flexion (F) for the thoracic (T) and lumbar (L) measurements. A comparison was made of these computed thoracolumbar angles (full flexion minus neutral, two-thirds flexion minus full flexion, and the full flexion minus two-thirds flexion positions) using paired samples t test with Bonferroni correction (p = 0.017), to determine whether these three thoracolumbar angle measurements were different.

#### Relative spinal measurements in thoracic versus lumbar spine

Descriptive statistics were used for comparison of the thoracic, lumbar and computed thoracolumbar measurements of the positions tested. Additionally, the use of a two (vertebral level of thoracic and lumbar) by seven (trials) mixed model repeated measures ANOVA indicated whether significant differences were present between the thoracic (T4) and lumbar (L3) angular measurements at each position tested. The use of an ICC (3, k) with the 95% confidence interval (CI) and standard error of the mean (SEM) indicated the reliability of the reposition trials from the thoracic (T4) and lumbar (L3) level measurements.

## Results

### Spine versus hip movement

Descriptive statistics (mean ± standard deviation) for the thoracolumbar angle for full flexion minus the neutral position was 65 ± 12.9°, two-thirds of full flexion minus the neutral position was 46 ± 12.4°, and full flexion minus two-thirds of full flexion position was 18 ± 8.4° (Table 3). Paired samples t-test with Bonferroni correction (p = 0.017) indicated the following comparisons between the thoracolumbar angles were all significantly different (p < 0.017); full flexion minus neutral versus two-thirds of full flexion minus neutral, two-thirds of full flexion minus neutral versus full flexion minus two-thirds of full flexion, and full flexion minus neutral versus full flexion minus two-thirds of full flexion. Comparison of the full flexion position angle to the two-thirds position angle at the thoracic and the lumbar levels should produce a value of 66.7%. The values produced were 70.5% and 63%, respectively and the mean of these values is 66.75% (Table 4).

**Table 3:** Descriptive statistics (mean, standard deviation, standard error, minimum and maximum values) in degrees for the angular measurements at the thoracic (T4) versus the lumbar (L3) levels.

	**Mean**	**Mean Difference T-L**	**Standard Deviation**	**Standard Error**	**Minimum**	**Maximum**
**Neutral 1 **						
T	12.38	-11.11	2.00	0.25	7.88	17.28
L	23.49		3.76	0.47	17.28	42.39

**Full**						
T	50.65	14.13	7.89	0.98	27.30	63.54
L	36.52		6.15	0.76	23.45	48.39

**Ref **						
T	39.37	7.67	7.00	0.87	23.56	52.4
L	31.70		4.56	0.57	23.39	42.34

**2/3 1 **						
T	38.75	7.41	6.51	0.81	21.38	51.68
L	31.34		4.48	0.56	22.14	41.88

**2/3 2 **						
T	38.82	7.34	6.72	0.83	20.96	52.53
L	31.48		4.67	0.58	22.14	42.22

**2/3 3**						
T	38.62	7.15	6.84	0.85	21.63	53.36
L	31.47		4.84	0.60	21.48	42.22

**2/3 4 **						
T	38.46	7.06	6.85	0.85	21.97	52.82
L	31.40		4.69	0.58	21.83	42.47

**2/3 5 **						
T	38.30	6.98	6.60	0.82	21.63	53.76
L	31.32		4.50	0.56	21.99	42.66

**2/3 6 **						
T	38.14	6.76	6.57	0.81	22.29	53.60
L	31.38		4.64	0.58	21.12	42.30

**2/3 7 **						
T	37.81	6.38	7.44	0.92	13.19	53.38
L	31.43		4.75	0.59	20.76	41.14

**Neutral 2**						
T	13.40	-10.24	2.66	0.33	9.12	26.74
L	23.64		2.89	0.36	16.9	32.68

T = Thoracic T4 Level

L = Lumbar L3 Level

Ref = Reference

2/3 = Two Third's Position

**Table 4:** Descriptive statistics (mean, standard deviation, standard error, minimum and maximum values) in degrees for the thoracolumbar angle computed from the thoracic T4 minus the lumbar L3 X and Y measurements for movement above the L3 level.

	**Mean**	**Standard Deviation**	**Standard Error**	**Minimum**	**Maximum**
**Neutral 1**	2.18	3.94	0.49	-6.16	14.13

**Full**	67.29	11.77	1.46	30.20	84.11

**Reference **	48.29	11.45	1.42	23.02	68.26

**Ref-2/3 1**	47.45	10.51	1.30	20.27	68.65

**Ref-2/3 2**	47.43	10.79	1.34	19.54	70.40

**Ref-2/3 3**	46.96	11.03	1.37	20.32	72.53

**Ref-2/3 4**	46.65	11.03	1.37	20.94	70.48

**Ref-2/3 5**	46.42	10.74	1.33	20.56	72.97

**Ref-2/3 6**	46.05	10.62	1.32	21.92	72.97

**Ref-2/3 7**	46.33	10.98	1.36	21.46	74.87

**Neutral 2**	3.16	4.09	0.51	-4.29	14.49

2/3 = Two Third's Position

Negative values indicate undershooting and positive values indicate overshooting the target position.

### Relative spinal measurements in the thoracic versus lumbar spine

**Figure 6:**
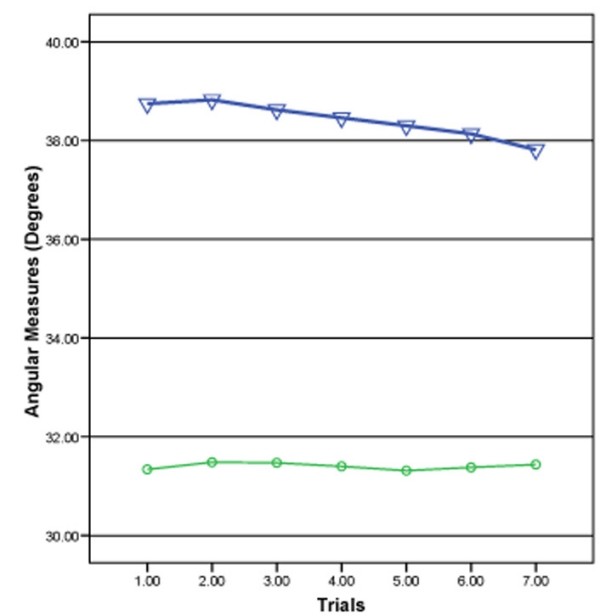
**Comparison of the angular measurements for trials 1–7 computed from the thoracic (T4 = triangles) and lumbar (L3 = circles) X and Y measurements**.

Comparisons of the mean angular changes at the T4 and L3 spinal levels (Table 5) revealed movement occurring at both the thoracic and lumbar levels. Comparison of the angular measurements calculated from the X and Y measures at each trial for the thoracic (T4) versus the lumbar (T3) level using a two (vertebral level) by seven (trials) mixed model repeated measures ANOVA produced no significant difference (F = 2.01, p = 0.13) for a vertebral level by trial interaction. Because of this non-significance, the use of the trials, as a main effect was validly used. The main effect for trials was not significant (F = 2.26, p = 0.10). The main effect for the vertebral level was significant (F = 48.20, p = 0.001). Graphical comparison (Figure 6) showed 1) no interaction between the thoracic and lumbar levels throughout all the seven trials, 2) the same process occurred throughout trials in the thoracic and lumbar spinal areas and 3) the thoracic measurements were seen as very different from the lumbar measurements. An ICC (3, 4) of 0.82 (95% CI, 0.73–0.88; 1.18 SEM) for the thoracic trials and 0.76 (95% CI, 0.65–0.84; 0.78 SEM) for the lumbar trials was found.

**Table 5:** Neutral, full flexion and the two-thirds (2/3) flexion position for thoracic T4 and lumbar L3 angle measurements including mean degrees ± standard deviation.

**Thoracic T4 Level**				**Lumbar L3 Level**			
Neutral	Full Flexion	Two-Thirds Flexion	Percentage of Full Flexion	Neutral	Full Flexion	Two-Thirds Flexion	Percentage of Full Flexion

12.38 ± 2.0	50.65 ± 7.89	39.37 ± 7.0	70.5	23.49 ± 3.76	36.52 ± 6.15	31.70 ± 4.56	63

## Discussion

### Spine versus hip movement

If the spine does not move during the protocol with the SRSD but instead rotation occurs at the pelvis on the femurs, no differences would be found for the computed thoracolumbar angle in the various positions tested. This angle represents movement occurring between T4 and L3 and is spinal movement. The significant differences (paired t tests) between the thoracolumbar angles (full flexion minus neutral versus two-thirds of full flexion minus neutral, two-thirds of full flexion minus neutral versus full flexion minus two-thirds of full flexion and full flexion minus neutral versus full flexion minus two-thirds of full flexion) provided evidence that the protocol used with the new SRSD allows movement in the spine. The descriptive data for the differences found in the thoracic and lumbar measurements also provided support (Table 3). Motion occurred within the lumbar and the thoracic spines. The first hypothesis that no difference would be found in the thoracolumbar angle between the various positions tested was rejected. The documented movement in the upper lumbar spine will be important for future use of the device for evaluation of treatment interventions and their proposed impact on spine reposition sense.

The measurement procedures used though did not allow determination of the amount of movement occurring at the pelvis on the femurs with this protocol. Previous literature has demonstrated that during forward bending, movement occurred through flexion of the lumbar spine and the pelvis on the femurs. The magnitude of the movement at the spine was greater than at the pelvis on the femurs, in the early stage of forward bending. In the final stage of forward bending, the relative contribution of the spine was reduced. [37-40] The contribution to forward bending from the lumbar spine was reduced in subjects with low back pain [39,40] as well as in subjects with back injury and asymptomatic subjects with a history of back pain. [37,41] Decreased range of hip flexion during forward bending of the trunk has been found in subjects with back pain. [37,38] Clinically, the evaluation of the lumbar spine, pelvis and hips, in subjects with back pain, should be considered.

### Relative spinal measurements in the thoracic versus lumbar spine

Because the spine did not move as a rigid body about the hips during the testing protocol, the second objective of where movement in the spine was taking place was addressed. The statistical findings using the mixed model repeated measures ANOVA and the graphical analysis (Figure 6) indicated the lumbar and the thoracic measurements were different from one another at all seven trials tested. The amounts of movement in the thoracic and lumbar spines are presented in Table 3. These data support rejection of the second null hypothesis (no difference would be found between the angular measurements taken at the T4 and L3 locations across the seven trials used in testing). Comparison of the subject's mean full flexion position value to the two-thirds position at the thoracic and the lumbar levels indicated the subjects were producing near to a two-thirds position in each area (Table 4). These thoracic and lumbar percentages of 70.5% and 63% respectively average to 66.75%, which was very close to a true two-thirds position.

The good ICC (3, k) findings for both the thoracic and the lumbar trials indicated good reliability. [42] The low SEM findings (0.78 and 1.18) associated with the ICCs (3,4) provided an estimate of the precision of the measurement. [43]

### Study Limitations

The results of this study are limited to healthy young adults. Additional testing with older subjects as well as subjects with spinal pathology needs to be completed to assess the use of the SRSD within these populations.

## Conclusion

Due to concerns with the new reposition sense device including 1) that the spine was moving as a rigid body rotating about the pelvis on the femurs during movement testing and 2) that movement was not specific to the lumbar spine, additional testing was completed. Spinal movement was found using the new SRSD methodology indicating the spine did not move as a rigid body. Movement was also found specific to the lumbar spine. This last finding will allow the device to be used to assess lumbar spine treatment intervention(s) suspected to impact spine proprioception which has not been previously assessed.

## Competing interests

The authors declare that they have no competing interests.

## Authors' contributions

All authors contributed equally to this work and read and approved the final manuscript.
